# Constrictive pericarditis in a patient with sinus venosus atrial septal defect and anomalous right upper pulmonary venous return

**DOI:** 10.4103/0974-2069.52820

**Published:** 2009

**Authors:** Santosh C Uppu, Sruti Chandrasekaran, Kiran K Mallula

**Affiliations:** Departments of Internal Medicine and Pediatrics, Albert Einstein Medical Center, Philadelphia, PA, USA

**Keywords:** Constrictive pericarditis, partial anomalous pulmonary venous return, sinus venosus atrial septal defect

## Abstract

This is a report of a 49-year-old male, who presented with typical signs and symptoms of constrictive pericarditis. He was diagnosed with sinus venosus atrial septal defect (ASD) and anomalous right upper pulmonary venous return during his adolescence, which was elected not to be repaired. During the attempted repair of the ASD it was noted there was a thick fibrous material covering the heart, which had progressed over time leading to frank constrictive pericarditis. His ASD spontaneously closed over time. There have been less than 10 cases reported with constrictive pericarditis of nonsurgical etiology in a patient with ASD, and none with sinus venosus ASD.

## INTRODUCTION

The combination of constrictive pericarditis (CP) and atrial septal defect (ASD) is rarely seen.[1] There have been less than 10 cases which have been reported in literature with no definitive causal relations.[1–5] The etiology of CP in the setting of sinus venosus ASD with partial anomalous pulmonary venous return (PAPVR) is still debatable. We report this rare case of CP with sinus venosus ASD and PAPVR.

## CASE REPORT

**Figure 1:**
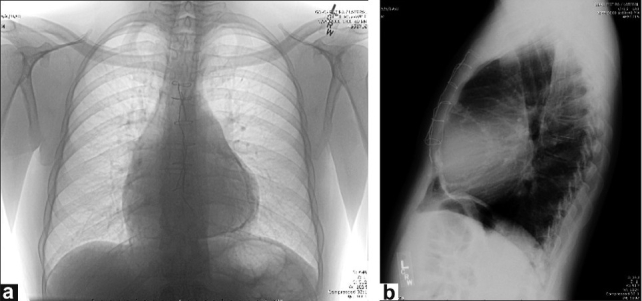
(a) Chest X ray PA view, inverted image (b) Chest X ray lateral view showing pericardial calcification

**Figure 2:**
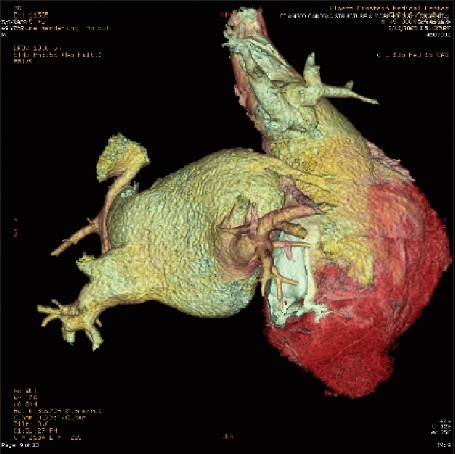
CT reconstruction- showing all four pulmonary veins. Right superior pulmonary veins can be seen draining in to superior vena cava

A 49-year-old African-American man was admitted due to increasing shortness of breath, abdominal distension, and swelling of both lower extremities for the past one month. Initially, he noticed abdominal distension followed by pedal edema, which then progressed. He was diagnosed with sinus venosus ASD at 15 years of age, as a part of a murmur workup, for which he underwent elective cardiotomy. The Mantoux test was negative. On opening the pericardium, the heart was noted to be enlarged and was covered with a tough, thick white fibrous material that was not adherent to the pericardium though it was adherent to the underlying epicardial tissue. This was confirmed as fibrous scarring by histopathology. The sinus venosus ASD was found to be 1-2 mm in size. Right (Rt) superior pulmonary vein was noted to enter the superior vena cava (SVC) 2.5-3 cm above Rt atrium. It was elected not to repair the ASD and the anomalous right upper pulmonary vein, as the surgeon felt that the cardiomegaly could be explained by the epicardial reaction and not by the presence of small ASD or PAPVR. The preoperative shunt was 1.6:1 by cardiac catheterization. After the surgery, he had several episodes of scar related atrial flutter requiring electrical cardioversions, Amiodarone, and anticoagulation. He underwent radiofrequency catheter ablation five years prior to his second surgery. Echocardiogram's done as a part of the workup for atrial flutter did not show ASD. He was a non-smoker and non-alcoholic. On examination, the vitals were normal and he was in sinus rhythm with a normal heart rate. A neck examination revealed a normal thyroid gland, no bruit, and elevated jugular venous pulse with a prominent ‘y’ descent. The Kussmaul's sign was negative. There was no pallor, cyanosis, clubbing or icterus and all peripheral pulses were equal. Cardiovascular examination revealed normal heart sounds; fixed splitting of the second heart sound with a pericardial knock. No murmurs or gallops were heard. Abdomen was distended with fluid and mild hepatomegaly was present. Extremities showed bilateral grade 2 pitting pedal edema. An electrocardiogram showed sinus rhythm with normal axis and a rate of 75. The chest X-ray [Figure 1a and b] revealed an enlarged heart with calcified pericardium. Liver function tests, renal function tests, hematological indices, and urine analysis were normal. Thyroid function tests revealed increased TSH and normal free T4. The workup for hemochromatosis was negative. An echocardiogram with color Doppler showed normal left ventricular ejection fraction (LVEF), with a thickened, calcified pericardium. Cardiac catheterization showed Q p: Qs of 1.8: 1 with normal pulmonary arterial pressures, normal cardiac output, mildly elevated Rt heart filling pressures, normal left heart filling pressures, and normal coronary arteries. A coronary CT angiogram [Figure 2] showed a thickened, heavily calcified pericardium. Two lower pulmonary veins and a left upper pulmonary vein were draining into the left atrium and two right upper pulmonary veins were anomalously draining into the superior vena cava at the junction of the right atrium; the patient also had right atrial and ventricular enlargement. The CT also showed a diffusely hyperdense liver and mild pulmonary interstitial infiltrates probably secondary to Amiodarone therapy. His Amiodarone was discontinued and heart failure was managed medically. A redo sternotomy with pericardial stripping and repair of anomalous pulmonary veins by creation of a baffle with bovine pericardium into the left atrium, and patch repair of SVC was performed at a later date. During the surgery, no ASD was found. The ASD might have spontaneously closed over the course of time. On histopathological examination, the atrial septum revealed muscle hypertrophy. The pericardium showed sclerosis, patchy chronic inflammation, calcification and ossification, and was negative for acid fast and gram stains. The patient's postoperative course was uneventful.

## DISCUSSION

Constrictive pericarditis of nonsurgical etiology with ASD is a rare co-occurrence.[1–5] CP with sinus venosus ASD and PAPVR has never been reported. This combination in our patient has been recognized incidentally. It is possible that CP and ASD could have different etiologies, and it is by chance that they have coexisted in our patient. On initial evaluation, he was noted to have both sinus venosus ASD and a fibrotic pericardium, with no clinical features of CP. As the patient grew older, his sinus venosus ASD closed spontaneously as was revealed by follow-up echocardiograms done as a part of the arrhythmia workup, after the initial surgery. It is likely that he manifested CP over time, which progressed to frank constriction along with pre-existing PAPVR. As his initial exploratory cardiotomy revealed an abnormal fibrotic pericardium, it was unlikely that CP might have resulted from surgery. CP in our patient might have been a slow ongoing process of unknown etiology. The combination of CP and ASD can be difficult to diagnose and can be overlooked.[4] It is extremely important to recognize the occurrence of these lesions, for appropriate and timely management.
